# Copolymerization of Mesoporous Styrene‐Bridged Organosilica Nanoparticles with Functional Monomers for the Stimuli‐Responsive Remediation of Water†

**DOI:** 10.1002/cssc.202001264

**Published:** 2020-08-10

**Authors:** Dennis Kollofrath, Marcel Geppert, Sebastian Polarz

**Affiliations:** ^1^ Institute of Inorganic Chemistry Leibniz-University of Hannover Callinstrasse 9 30167 Hannover Germany; ^2^ Department of Chemistry University of Konstanz Universitätsstrasse 10 78457 Konstanz Germany

**Keywords:** core-shell particles, drug removal, mesoporous materials, organic-inorganic hybrids, water

## Abstract

For every mass product, there are problems associated with the resulting waste. Residues of hormones in urine cannot be removed sufficiently from wastewater, and this has undesired consequences. An ideal adsorbent would take up the impurity, enable a simple separation and recyclability. Polymer colloids with high affinity towards the drug, accessible porosity, high surface area, and stimuli‐responsive properties would be candidates, but such a complex system does not exist. Here, porous vinyl‐functionalized organosilica nanoparticles prepared from a styrene bridged sol‐gel precursor act as monomers. Initiation of the polymerization at the pore walls and addition of functional monomers result in a special copolymer, which is covalently linked to the surface and covers it. An orthogonal modification of external surface was done by click attachment of a thermoresponsive polymer. The final core‐shell system is able to remove quantitatively hydrophobic molecules such as the hormone progesterone from water. A change of temperature closes the pores and induces the aggregation of the particles. After separation one can reopen the particles and recycle them.

## Graphical abstract

**Drugs are out**: The internal surfaces of mesoporous organosilica nanoparticles are designed for the quantitative uptake of drugs from water. This is achieved by decoration with functional polymers by copolymerization with the pore walls. A shell of a thermoresponsive polymer was attached at the exterior of the particles. This leads to a closure of the pores at higher temperature and aggregation of the particles, which facilitates their separation. Re‐opening and release of the entrapped molecules allows to regenerate the adsorbent.

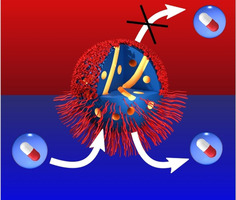

## Introduction

An obvious advantage of nanostructured matter is the maximization of the interfacial area per volume or per mass. A high specific surface area is of pivotal importance for numerous applications for example in catalysis,^1^, ^2^, ^3^ sensing,^4^, ^5^, ^6^ and adsorption.^7^, ^8^, ^9^ But a high surface area alone is not enough, and desired properties are only realized if a maximum density of active, functional groups exists at the accessible interface. The range of possible functionalities is richest for molecular organic compounds. Thus, the ideal scenario would be to shape a functional organic into a nanostructure with high surface area as it is present in porous solids. However, due to the weak intermolecular forces in organic systems the open porosity with a high surface area vanishes quickly in the thermodynamic equilibrium. Stronger interactions which lead to a stable material backbone can be secured by the introduction of an inorganic matrix. This highlights the importance of organic‐inorganic hybrid materials whose prototype is given by organosilica materials.^10^, ^11^

The number and extension of review articles which have been written on (porous) organosilica materials documents a huge variety of functional groups incorporated in such hybrid structures.^12^, ^13^, ^14^ These include thiols,^15^ amines,^9^ carboxylic acids,^16^ azides,^17^ halides^18^ and alkynes.^19^ Due to its versatility, the vinyl function takes on a special role among the organic modifications of silica surfaces. It can be easily transformed into alcohols by hydroboration^20^ or further functionalized by using established thiol‐ene click chemistry.^21^, ^22^, ^23^, ^24^ Furthermore, vinyls are key constituents in many monomer molecules and therefore their presence grants access to a high variety of organic‐polymer hybrid materials.

The vinyl functionality can be introduced into an organosilica material by co‐condensation^25^, ^26^, ^27^, ^28^, ^29^, ^30^ or by grafting^31^, ^32^ of vinyl‐containing compounds (e. g., vinyltriethoxysilane). However, the degree of functionalization in the resulting materials is relatively low and in case of grafting the functionalization can be inhomogeneous.^33^ Note that regarding post‐grafted materials, it is difficult to secure maximized organic content along with a high and accessible porosity. This is due to significant decrease of the surface area after the grafting process. These issues can be avoided by using bridging sol‐gel‐precursors of the X_3_Si−R‐SiX_3_ type (X=alkoxide, R=functional organic group).^34^ Materials prepared by using such precursors are outstanding amongst organosilicas. They offer precise tuneability of both the strength of the inorganic backbone as well as of the type and number of functional groups. The density of the organic groups is maximized (RSi_2_O_3_) when the precursor is used undiluted.^35^, ^36^, ^37^ While bridging precursors containing a non‐terminal double bond like 1,2‐bis‐(triethoxysilyl)ethylene are well known in literature,^22^, ^38^, ^39^, ^40^, ^41^ corresponding nanoporous materials with R containing a vinyl group are not yet discussed.^37^ Because the systems mentioned previously lack a terminal vinyl group, it is not surprising they have not been used as monomers or for the copolymerization of the surfaces of the silica with organic monomers. However, the usage of the pore system of silica‐based materials as a confined space for polymerization is known in literature. Choi et al.^42^ investigated the polymerization of chloromethylstyrene inside of the pores of a SBA‐15‐like material. By combining the wet‐impregnation method with free‐radical polymerization they were able to create poly(chloromethylstyrene) inside of a SBA‐15 material. Comotti et al.^43^ used the pore system of an organized organosilica as a reaction vessel for the confined polymerization of acrylonitrile followed by the thermal conversion to a carbon/silica nanocomposite. The polymer was not covalently linked to the silica network in neither of the mentioned cases, leading to a reduced stability of the material. Polymer attachment was achieved by Jadhav et al.^44^ by using 3‐(methacryloxypropyl) trimethoxysilane (MPS) as a graftable vinyl source followed by free radical polymerization of *N*‐isopropylacrylamide (NIPAM) in the pore channels. An alternative approach was mentioned by Kruk et al.^45^ who used atomic transfer radical polymerization (ATRP) for in situ polymerization of polystyrene and PMMA in the pore system of SBA‐15‐like material. Prior to polymerization they grafted an ATRP initiator to the free silanol groups on the pore wall, resulting in covalently linked polymer brushes. This method is also known as surface‐initiated polymerization.^46^ All those procedures are based on either grafting the vinyl functionality, initiator or even prefabricated polymer chains to the silica network, leading to the well‐known and previously mentioned issues.^47^, ^48^ The inhomogeneous distribution of the functionalization, low grafting yields and pore blocking are the most important.^33^, ^49^

The targeted synthesis of porous organic‐inorganic hybrid systems poses a challenge even for bulk materials. Reaching the nanoscale for such a material is even more complex.^50^ However, significantly higher surfaces and colloidal stability in solutions offer an incentive to overcome the challenges of nanoparticle synthesis.^50^, ^51^, ^52^, ^53^ An excellent overview was published by Yamamoto and Kuroda.^54^

The Stöber method and its variations are the most commonly used procedures for the generation of nanoporous organosilica particles (NOPs). It seems the results of the Stöber method become worse, the more a precursor differs from the well‐known behavior of Si(OEt)_4_. Therefore, relatively few papers on using undiluted bridging precursors X_3_Si−R‐SiX_3_ for the preparation of NOPs are present, and the reader is referred to the review articles by Croissant et al.^50^ and Chen and Shi.^11^ Most publications cover systems with quite simple bridges or systems where the bridged precursor is diluted with an substantial amount of Si(OEt)_4_.^50^, ^53^, ^55^, ^56^, ^57^ We know from our own research how difficult it is to obtain NOPs with well‐structured pore‐systems out of functional X_3_Si−R‐SiX_3_ precursors.^15^, ^58^, ^59^ There are very few exceptions such as the report by van der Voort and co‐workers,^60^ where allyl‐modified tris(diethoxysila)cycloheaxane precursor mixtures are synthesized and used for the generation of NOPs for chromatography. Organosilica nanoparticle synthesis using vinyl containing bridging precursors are not yet discussed in literature.

Higher complexity and, thus, higher functionality are possible, if the internal regions of the particles are chemically different to the external surfaces. Such systems are often referred to as core‐shell structures, and the architecture can either be introduced by growing a chemically different shell around a core or by locally restricted multi‐functionalization of the same material. For the latter option, the most common technique is a preservation of the pore template,^61^ which allows a locally restricted modification of the outer surface of the particles. Afterwards, the template is removed, and a different modification is performed inside the pores of the particles.^62^ Another approach is to use orthogonal, highly chemo‐selective reactions to achieve spatially resolved functionalization. Using this concept, Li et al. obtain bifunctional core‐shell nanoparticles by combination of azide‐alkyne and thiole‐ene surface click reactions.^63^ These multifunctional materials can be used in a very wide range of applications such as catalysis of tandem reactions,^1^ the generation of amphifunctional properties,^62^, ^64^ or more advanced delivery systems such as SiRNA carriers presented by Bein and co‐workers.^65^

Another application area in which these materials have proven themselves is water treatment. Water treatment is an urgent issue as the demand for clean drinking water is increasing due to population growth, while at the same time millions of tons of pollutants are discharged into waters each year.^66^ These can be of industrial, civilizational or natural origin. Materials chemistry has already developed systems based on functionalized silica nanoparticles for removing heavy metals,^67^, ^68^, ^69^ pesticides,^70^ dyes^62^, ^71^, ^72^, ^73^ or other organic pollutants.^74^, ^75^ Hormones are a special type of organic pollutant as most of them are part of the endocrine disrupting chemicals, meaning that even the smallest quantities are sufficient to have an influence on the human body. Therefore, systems for the purpose of removing hormones from wastewater require a high adsorption affinity to the hormone to remove it as quantitatively as possible. Carvalho et al. used β‐cyclodextrin surface‐functionalized silica particles to entrap methyltestosterone inside of β‐cyclodextrin chains.^76^ Gao et al. tackled the issue by phenyl modification of mesoporous silica particles, making the pore environment more hydrophobic, which lead to an increased uptake of different estrogens.^77^

**Scheme 1:**
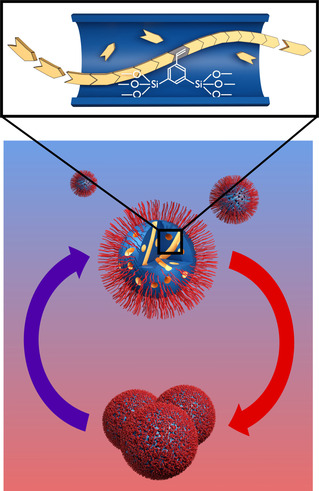
Top image: Cross‐Section of the internal regions of one pore demonstrating the copolymerization process between organic monomers and the organosilica surface. Bottom: The exterior surfaces of the organosilica particles can be modified independently from the pores with a different polymer. The core (pore system) is functionalized via free‐radical polymerization whereas the shell is functionalized using thiol‐ene click chemistry. If the exterior polymer is thermoresponsive, the particles do not only aggregate and deaggregate depending on temperature, the pores also open and close.

In this paper, we report the synthesis and characterization of a bridged sol‐gel precursor containing a terminal vinyl group and its usage in the nanoparticle synthesis. The precursor solves the issues mentioned above by intrinsically containing a vinyl group leading to particles with an extraordinary high degree of functionalization. Surface area, pore diameter and pore volume were optimized for the later functionalization. We describe a method for the selective functionalization of the pore system by free radical polymerization with different monomers. The particles act simultaneously as a structural backbone and a co‐monomer leading to the formation of a copolymeric structure. 2,3,4,5,6‐pentafluorostyrene is among others used as a monomer to make the pore environment strongly hydrophobic. By using thiol‐ene click chemistry for the functionalization of the outer surface of the particles, we obtained multifunctional core‐shell particles. Poly(*N*‐isopropylacrylamide) (PNIPAM) was used as the shell functionalization leading to thermo switchable pore accessibility and particle aggregation. The core‐shell‐architecture of the functionalized NOPs is characterized in detail, and stimuli‐responsive properties are investigated by the separation of hydrophobic impurities represented by Solvent Yellow 14 as a organic model dye and progesterone as a hormone/pharmaceutical from aqueous solutions (Scheme 1).

## Results and Discussion

### Styrene‐bridged NOPs

**Scheme 2:**
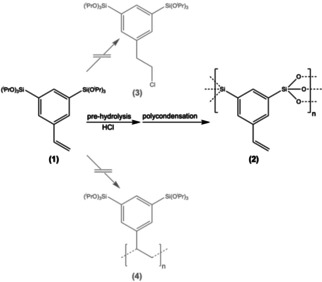
Desired (black) and unwanted (grey) pathways of the styrene‐bridged sol‐gel precursor to different reaction products.

For the preparation of nanoporous organosilica particles (NOPs) composed of bridging vinyl monomer moieties, we have used 1,3‐bis(tri‐iso‐propoxysilyl)‐5‐styrene (**1**); see Scheme 2. The synthesis was presented by us as a proof‐of‐concept before,^18^ and was improved here (see the Experimental Section). Unambiguous characterization of the precursor is summarized in the Supporting Information Figure S1. Although the sol‐gel precursor **1** is obviously a good entry point into polymer‐organosilica hybrid materials, this possibility was not explored in our previous work at all.^18^

There are three difficulties encountered, which had to be mastered. (i) The hydrolysis of the isopropoxysilyl groups is typically performed under acidic conditions (pH=0). Unfortunately, HCl can react with the vinyl group via addition (Scheme 2). (ii) Because **1** is a monomer, it can polymerize via thermal initiation. This event is obviously also undesired as the vinyl groups are required later on (Scheme 1). (iii) There is a special electronic situation at the silyl groups due to direct attachment the styrene moiety, which impedes hydrolysis. As a result, standard conditions lead to rather undefined product shapes which cannot be described as NOPs.

The hydrolysis kinetics were studied in detail and the results are shown in Supporting Information Figure S2. It can be observed that hydrolysis is very fast at standard pH‐value (pH 0) but there is a significant loss of vinyl groups (Scheme 2: **1**→**3**). As expected, hydrolysis rate becomes slower at higher pH (pH 1), but with increasing pH‐value the number of remaining vinyl functionalization is increased as well. Therefore, it is very important to precisely control both pH and hydrolysis time (see the Experimental Section). It was found pH=2 is ideal, because the vinyl groups are not yet attacked by HCl while the hydrolysis occurs in an acceptable time frame (Figure S2).

A further issue are the alkaline conditions that are required to initiate the Stöber‐type growth of spherical silica particles. Therefore, we established a two‐step process. After hydrolysis is performed as described above, polycondensation was done in a buffer solution at pH=9.4 (see the Experimental Section). Two types of surfactants were used as a mixture, cationic cetyltrimethylammonium bromide (CTAB) and non‐ionic PEO (polyethylene oxide)‐based surfactants. Although the surfactants form mixed micelles and therefore both contribute to the formation of the pore system, the influence of Pluronic P123 on the pore system is significantly greater than that of CTAB.^78^, ^79^ The main role of CTAB is to stabilize the colloids formed in solution and to ensure a spherical particle shape.^80^

**Figure 1:**
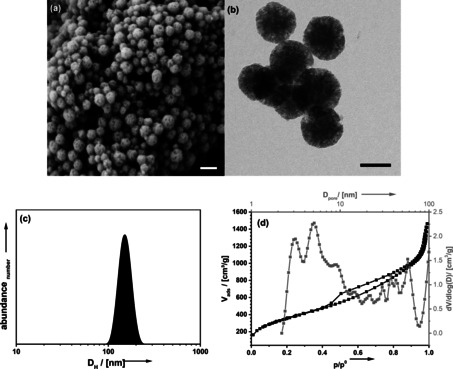
SEM (a; scalebar=200 nm), TEM (b; scalebar=100 nm) micrographs and DLS‐derived particle‐size distribution function (c) of NOPs prepared using Pluronic‐P123 as a template (P5). (d) N_2_ physisorption isotherm (black) and pore‐size distribution function (grey) of the material.

Particles that are irregular in size and shape with a pore diameter of 2 nm (Table 1; P1) are obtained. However, a sufficiently large pore size is crucial for the success of the later functionalization of the pore system as it ensures the homogeneous diffusion of the reactants. To overcome this issue, the pore size can be increased by adding a hydrophobic swelling agent to the condensation reaction.^81^ The degree of enlargement is controlled by the solubility of the swelling agent in the micelles of the template. For templates consisting of ethylene oxide and propylene oxide (common Pluronic surfactants), alkyl‐substituted benzene derivatives have proven to be appropriate. Table 1 shows the influence of different swelling agents on the pore system of styrene‐bridged NOPs (P1–P5). The particles synthesized using 1,3,5‐triisopropylbenzene (TIB) as swelling agent fulfill the requirements for a subsequent functionalization (P5). The particles are isotropic in shape (Figure 1) and typically have diameters of the order of 150 nm according to dynamic light scattering (DLS), scanning electron microscopy (SEM) and transmission electron microscopy (TEM). The porous nature of the particles can be observed from SEM and TEM images. The pore‐system seems quite irregular which is confirmed by the pore‐size distribution determined from N_2_ physisorption measurements. The broad range of pore diameters reaches from *D*_H_=3–12 nm while the specific surface area *A*_BET_ (BET=Brunauer, Emmet, Teller) amounts to 1306 m^2^ g^−1^.

**Table 1:** Optimization of the pore diameter by variation of reaction parameters.

Sample^[a]^	Template^[b]^	Swelling agent^[c]^	n (CTAB)^[d]^ [mmol]	T^[e]^ [°C]	V_p_^[f]^ [cm^3^ g^−1^]	A_p_^[g]^ [m^2^ g^−1^]	d_p_^[h]^ [nm]	Particle diameter^[i]^ [nm]
P1	Pluronic P123	–	0.11	25	0.84	975	2.1	103
P2	Pluronic P123	TMB	0.11	25	1.60	1239	2.8	77
P3	Pluronic P123	cyclohexane	0.11	25	0.86	738	2.5	86
P4	Pluronic P123	pentanol	0.11	25	0.49	888	2.1	54
P5	Pluronic P123	TIB	0.11	25	2.00	1306	5.1	150
P6	Pluronic L121	TIB	0.11	25	1.38	1075	4.2	126
P7	Pluronic F127	TIB	0.11	25	0.55	406	2.9	127
P8	Pluronic 31R1	TIB	0.11	25	1.40	1413	4.2	570
P9	Pluronic P123	TIB	0.11	10	1.01	795	4.7	132
P10	Pluronic P123	TIB	0.11	50	1.42	1259	3.0	37
P11	Pluronic P123	TIB	0.03	25	0.49	875	2.1	no particles
P12	Pluronic P123	TIB	0.30	25	1.23	1197	2.8	109

[a] All particle syntheses were performed using 100 mg of precursor; the procedure corresponds to the synthesis presented in the Experimental Section. [b] Template used in the particle synthesis for generating the pore system. [c] Hydrophobic additive for enlargement of the pore diameter; TMB is 1,3,5‐trimethylbenzene, TIB is 1,3,5 triisopropylbenzene. [d] Amount of CTAB in the reaction solution for the condensation reaction. [e] *T* is the reaction temperature during condensation reaction [f] *V*_p_ is the pore volume calculated by the NLDFT method. [g] *A*_p_ is the BET surface area. [h] *d*_p_ is the pore diameter determined by the NLDFT‐method. [i] Particle diameter determined from TEM images.

The structure of the pore‐system can be improved by using an alternative template (Table 1; P6–P8). By using Pluronic 31R1 (P8) the pore system gets much more uniform in size (Figure S2e). However, the pore‐size is too small (*D*_pore_=4 nm) and the particles are irregular in shape and size. The smaller pore size would prove disadvantageous for the intrapore polymerization reaction planned later. A change of the reaction temperature during the condensation reaction did not lead to a higher order at constant pore volume either (Table 1, P9–P10). Also, the variation of the Pluronic P123 to CTAB ratio had no positive effect on the pore and particle size (Table 1, P11–P12). However, the absence of particles at a reduction of the CTAB content supports the hypothesis that CTAB is mainly responsible for particle formation, while Pluronic P123 has more influence on the pore system (P11). Therefore, we have worked with the material prepared using Pluronic‐P123 (Figure 1; Table 1, P5) as it has the higher pore volume and the larger pore size.

The vinyl groups must be proven to be intact for the use of **2** in intrapore polymerization reactions. The material was analyzed spectroscopically and by thermogravimetric analysis. The data are summarized in Supporting Information Figure S3. A very powerful tool to determine the existence of the vinyl groups is FT‐Raman. The vibrational band at 1630 cm^−1^ is characteristic for the vinyl group. A comparison to the band belonging to the aromatic ring (1000 cm^−1^) indicates that most of the vinyl groups are still intact. The same conclusion can be drawn from comparing the vibrational band at 1630 cm^−1^ of **2** with the one from the molecular precursor (at the same wavenumber). The existence and amount of the vinyl groups is finally confirmed by ^13^C‐solid‐state NMR spectroscopy. The signals of the phenyl ring (122–140 ppm) are distinct from the vinyl signals (115 ppm). Evaluating the signal intensities demonstrates that approx. 90 % of the vinyl groups are still present. By dissolving **2** in a solution of sodium hydroxide in deuterium oxide it is possible to use ^1^H‐NMR technique to determine the remaining vinyl group content in the sample and substantiate the findings from solid‐state NMR spectroscopy. By comparing the aromatic signals with those of the vinyl group, the remaining vinyl content can be determined. The NMR spectrum (Supporting Information S3c) shows that 98 % of the vinyl groups are still intact. Taking the resolution of the ^13^C‐MAS‐NMR in account, the results of both measurements fit together. According to thermal gravimetric analysis (TGA) thermal elimination/oxidation of the styrene moiety occurs at *T*>265 °C. Also, the mass lost (42.7 %) fits very well to the mass‐loss expected for the conversion of an organosilica Ph−CH=CH_2_Si_2_O_3_ to 2xSiO_2_ (Δ*m*_th_=42 %). All of the latter results prove that the undesired pathways discussed in Scheme 2 were avoided. The material is perfectly suitable for copolymerization with molecular monomers.

### Copolymerization of functional monomers

**Scheme 3:**
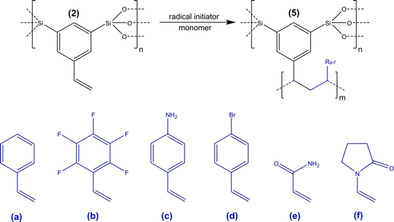
Copolymerization vinyl‐functionalized organosilica NOPs with different functional monomers.

As shown in Scheme 1 our next goal is the copolymerization of the vinyl groups in **2** with functional, molecular monomers (Scheme 3).

**Figure 2:**
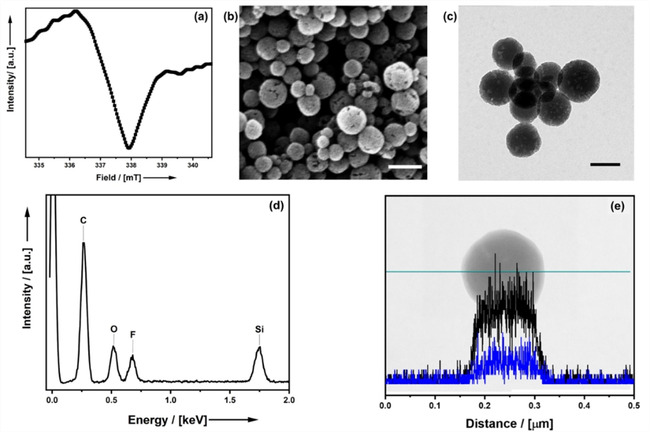
(a) EPR spectrum of **2** after treatment with AIBN. (b) SEM (scalebar=200 nm), (c) TEM (scalebar=100 nm), (d) EDX spectrum and (e) EDX line‐scan (black=Si, blue=F) of **3 b** after intraparticle copolymerization with pentafluorostyrene as a monomer (see Scheme 3).

The obvious choice of monomers are styrene derivatives as their polymerization products belong to the most established and well‐known polymers. A major challenge of this task is to avoid the polymerization of the monomer outside of the NOPs, which would lead to the formation of a polymer block with imbedded particles (see Supporting Information Figure S4). Therefore, incipient wetness impregnation was used to confine the initiation of the polymerization process to the mesopores. In order to prove that this method is suitable **2** was treated with a solution of the radical initiator AIBN (azobisisobutyronitrile). The NOPs were then separated from the solution, washed, and probed by electron paramagnetic resonance (EPR) spectroscopy. A very strong EPR signal is present at a *g* factor of 2.006 (Figure 2a), a value which supports the conclusion that the observed signal corresponds to an organic radical. The extremely broad signal can be explained by strong dipolar coupling of the radical species that are immobilized at the pore surfaces and in close proximity to each other.

Styrene is not ideal for proving the success of the intraparticle polymerization process, because it is chemically similar to the organic part of the precursor material. Therefore, we have selected alternative monomers with elements, which are clearly distinguishable by energy dispersive X‐ray (EDX) or solid‐state NMR spectroscopy. We elaborate our results here for pentafluorostyrene as a monomer, but analogous experiments additionally were performed with a variety of monomers (Scheme 3, see also Supporting Information S6) making this a highly adaptable system. SEM (Figure 2b) shows that the particles keep their shape while no polymer is observable outside of the particles (compare to Supporting Information Figure S4).

**Figure 3:**
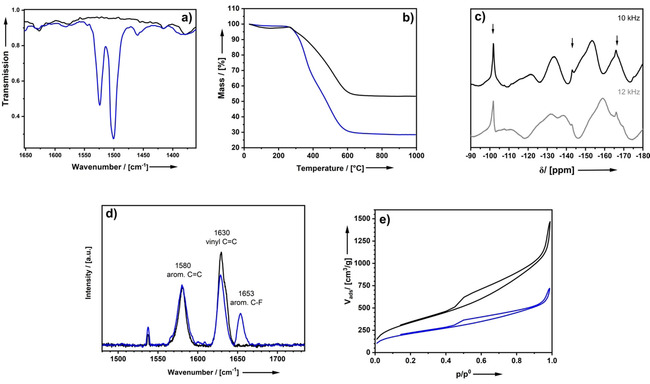
FTIR spectra (a), TGA (b), ^19^F solid‐state NMR spectra at different rotation speeds to eliminate ration sidebands (c), FT‐Raman (d) and N_2_‐physisorption isotherms (e) of **2** (black) compared to **3 b** (blue) after intrapore polymerization of 2,3,4,5,6‐pentafluorostyrene.

At a closer inspection of the image and comparison to Figure 1a (see also Electronic Supplementary Information Figure S5) it seems the imaging contrast between pores and material is less pronounced after the polymerization process which indicates a filling of the pores with polymer. A similar effect may be observed in TEM (Figure 2c, Figure 1b, Supporting Information Figure S5). EDX line‐scan condenses the hint by showing a distribution of fluorine over the whole particle (Figure 2e). Unambiguous proof of a formation of poly‐2,3,4,5,6‐pentafluorostyrene inside the pores of the NOPs, comes from the application of further analytical methods. Fig 3a shows FTIR spectra before and after polymerization of pentafluorostyrene. Two additional bands appear at 1500 cm^−1^ and 1524 cm^−1^ after polymerization which can be assigned to the C−F stretching vibrations.^82^, ^83^ Because the pores are now filled with polymer, whereas the number of siloxides remains constant, it is no surprise that a higher mass loss is measured by TGA (Figure 3b). The difference in the TGA traces of **5 b** and **2** shows that after polymerization roughly 25 % of the sample mass consist of the organic polymer. ^19^F‐MAS‐NMR spectra shown in Figure 3c further confirm that the functionalization with poly‐2,3,4,5,6‐pentafluorostyrene is successful. The spectrum was measured at two different rotation speeds to eliminate rotational sidebands. Signals that do not belong to the rotational side band remain at the same ppm value at both rotation speeds. The three detected signals can be assigned to the different Ar−F species.^84^ The Raman band at 1630 cm^−1^ decreased in intensity (Figure 3d) after the polymerization reaction while there are still significant amounts of unreacted vinyl groups left. This scenario is perfectly plausible and expected if a part of the vinyl groups of the bridging styrene moiety in the pore‐walls have become part of the growing polymer while the vinyl groups on the surface stay untouched. The appearance of the C−F vibration band at 1653 cm^−1^ wavenumbers indicates that a certain amount of functionalization occurred successfully.^85^ The reduction of the pore volume determined from N_2_‐physisorption measurements from 2.0 cm^3^ g^−1^ to 1.0 cm^3^ g^−1^ given in Fig 3e additionally shows modification of the particles’ pore system. However, the fact that the material still features open porosity, and the pore‐size has shifted to lower values by approx. 2 nm (Supporting Information Figure S5), demonstrates that the polymer does not clog the pores. Instead, it is covering the pore‐walls because the attachment is conducted via the styrene moieties of the organosilica. Our goal (Scheme 1) to create a copolymer built from organosilica material and the organic polymer was successfully achieved.

### Core‐shell functionalization

An enormous advantage of using the styrene‐bridged precursor **1** is the high amount of accessible vinyl groups in the system. As we restricted the polymerization to the inner surface of the NOPS, the untouched vinyl groups on the particle's surface can further be functionalized. This way, we aim to obtain multifunctional core‐shell NOPs. There are two major challenges that need to be addressed. (i) A further functionalization of the pores surface must be avoided. (ii) The functionalization should be homogeneous, as a later purification of the particles is difficult.

**Scheme 4:**
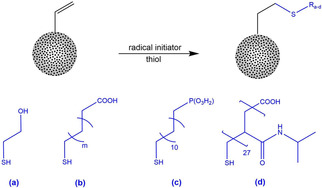
Thiol‐ene click chemistry on the particle surface with different functional thiols.

Thiol‐ene click chemistry meets both requirements as it is a very fast and quantitative method. Furthermore, a vast number of functional groups can be introduced by commercially available thiols (Scheme 4). To circumvent specifically issue (i), polymer chains with a chain length larger than the pore diameter of the particles are used so that the modification of the inner surface is avoided. The combination of free radical polymerization and thiol‐ene click chemistry is a powerful tool to create a highly adaptable multifunctional core‐shell system. Here, we use thiol‐capped, thermoresponsive poly(*N*‐isopropylacrylamide) (PNIPAM) chains for the shell functionalization, as we wanted to equip the system with stimuli responsive properties. When heated above 32 °C PNIPAM surpasses its lower critical solution temperature (LCST) and undergoes a reversible phase transition from a swollen hydrated state to a shrunken dehydrated state, losing about 90 % of its volume. This opens the possibility to regulate the pore accessibility with the temperature.

**Figure 4:**
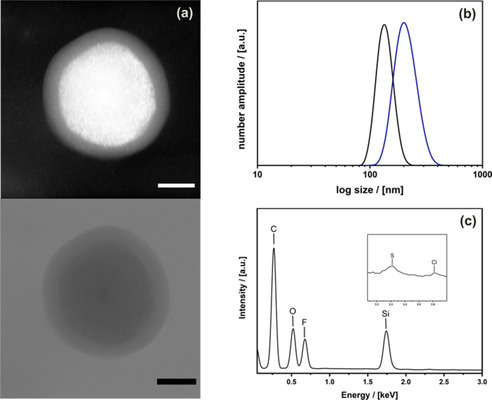
(a) HR‐TEM (bright and dark‐field; scalebar=50 nm) micrographs, (b) DLS‐derived particle size distribution function of a dispersion of the NOPs in water at 25 °C (blue) and 40 °C (black), and (c) EDX of core‐shell NOPs with pore surfaces modified with 2,3,4,5,6‐pentafluorostyrene and shell decorated with PNIPAM.

High resolution (HR)‐TEM imaging of the resulting material (Figure 4a) shows a homogeneous corona around the particles indicating a successful functionalization. The thickness of the corona is around 20 nm and fits well to the calculated, elongated chain length of the PNIPAM chain (*M*_n_=3.000 g mol^−1^). Switchability of the PNIPAM chains is shown by DLS measurements as the diameter of the particles shifts, depending on whether the PNIPAM chain is hydrated or collapsed (Figure 4b). The hydrodynamic radius decreases by 50 nm from 190 nm at 25 °C to 140 nm at 40 °C, which is in good agreement with the TEM images. N_2_ physisorption measurements (Supporting Information Figure S7) show a decrease of the BET surface area and the pore volume of the NOPs after functionalization. The BET decreases from 989 to 449 m^2^ g^−1^, the pore volume from 1.57 to 0.39 cm^3^ g^−1^, demonstrating that the collapsed PNIPAM chains can shield most of the pore system. Because the PNIPAM chains are attached at the outside of the particles, it can be secured they do not clog the internal pores. Finally, the detection of both fluorine and sulfur in the EDX shows that both the inner as well as the outer surface were successfully modified. (Figure 4c). The synthesis of the material (Scheme 1) was performed successfully and the use of the material can be tested.

### Release experiments and water remediation

**Figure 5:**
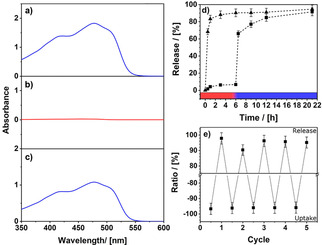
UV/Vis spectra and photographic image of the initial NOPs/SY14 mixture in water/ethanol dispersion (a), after uptake of the dye in the pores of the material (b), and after release of the dye (c); (d) time‐dependent study of SY 14 release using PNIPAM core‐shell‐functionalized NOPS (squares) and a reference without PNIPAM shell (triangles). In the case of PNIPAM‐functionalized NOPS, pores were opened after 6 h by temperature decrease. 100 % is referenced to the amount confined to the pores in the first run (e). Adsorption and release cycles of SY14 into PNIPAM‐functionalized core‐shell NOPs.

To show that not only the length of the PNIPAM chains is switchable but also the accessibility of the pores, we performed uptake and release experiments with the dye SY14 (Solvent Yellow 14), which represents a substitute for any hydrophobic organic contaminant. Figure 5a shows a photography of the particles dispersion in water containing SY14. The particles are well dispersed, and the color of the dispersion is characteristic for SY14. The change of the temperature to 40 °C leads to a collapse of the PNIPAM groups. This reduces the colloidal stability of the particles and closes the pores (Scheme 1). The particles can be separated from the solution and the dye is trapped inside the pores of the organosilica particles. The extent of the uptake of the dye can be determined best by using UV/Vis spectroscopy. The absorption spectrum of the dissolved dye before addition of the particles is shown in Figure 5a. The absorption at *λ*_max_=481 nm is 1.86 OD. Another UV/Vis spectrum was measured after separation of the dye‐filled particles (Figure 5b) from the solution. The residual solution shows no absorbance signal, which demonstrates the quantitative removal of SY14. Afterwards, the dye is released from the NOPs by decreasing the temperature again, and the resulting spectrum is shown in Figure 5c. As expected, the signals of SY14 appear again. The absorption value is lower, which means that some part of the dye remains in the particles despite opening of the pores or has been washed out prior to release since not every pore is blocked by the collapsed PNIPAM chain. As the absorbance is directly proportional to the dye concentration (path length is kept constant) the uptake capacity of the particles can be determined by using the Beer‐Lambert law (Supporting Information Figure S8a). A capacity of 69.4 mg per g of particles is found, which is in agreement with other dye adsorbents known to literature.^73^, ^86^, ^87^, ^88^, ^89^, ^90^ The procedure and necessary calculations are described in the Experimental Section and Supporting Information (Figure S8). To proof that the PNIPAM functionalization is responsible for the stimuli responsive dye release we performed time dependent release experiments of particles with and without PNIPAM shell functionalization. Both particle types were loaded with SY 14 and the release was observed by UV/Vis spectroscopy (Fig 5d). It becomes apparent that the dye uptake is not dependent on the functionalization of the shell (Supporting Information Figure S8c). Both materials absorb the dye completely. A difference can be observed in the release process of the dye. First, both particle sets were stirred at 40° C for several hours to investigate whether an unintentional release (leakage) occurs while the pore system is closed (Fig 5d, red region). While in the case of the unfunctionalized particles the dye is already rapidly and uncontrolled released, there is only a very limited dye release of the PNIPAM‐functionalized particles. Next, the temperature of the dispersion was decreased opening the pore system and leading to further release of dye (Fig 5d, blue region). Since the unfunctionalized particles have already released almost all the absorbed dye, the change in release is negligible. For the PNIPAM‐functionalized particles a strong increase of the released quantity is detectable after the trigger. This proves the stimuli responsive release. In total, 96 % of the adsorbed dye is released again showing that this system can compete with the current literature.^91^ Both, functionalized and unfunctionalized particles release the same amount of dye indicating no negative interactions of the PNIPAM shell with the adsorbed molecules. Since reusability plays a major role in the context of sustainability, the particles were examined regarding their recyclability. Five uptake and release cycles of dye were performed, and both the capacity and the high release rate are maintained (Figure 5e).

To show that the PNIPAM‐functionalized core‐shell NOPs can also adsorb compounds, which truly represent problematic contaminations in wastewater, we selected progesterone (P4) as a hormone and representative for residues of pharmaceuticals originating from oral birth control in urine. Therefore, we have treated an aqueous solution of P4 with NOPs followed by a temperature induced closure of the pores

**Figure 6:**
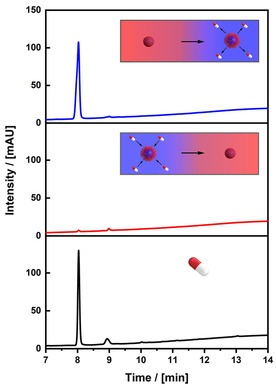
Chromatogram of an aqueous progesterone solution before treatment with our core‐shell particles (black), after treatment with the multifunctional core‐shell NOPs for 2 h (red) and the release in ethanol/water mixture at 20 °C (blue).

The particles were separated, and later, trapped progesterone can be released again by reopening the pores. The entire process was monitored by high performance liquid chromatography (HPLC) chromatography (Figure 6). The signal of P4 in the initial solution (prior to addition of the particles) can be observed at a retention time of 8.03 s. The significant reduction of this signal later on shows a significant uptake of P4 by the particles. Quantitative evaluation of the signal indicates that the uptake is almost complete (less than 0.02 % remaining in solution). Afterwards, pores are reopened by temperature adjustment and P4 is released. Consequently, the signal for P4 reoccurs and P4 is released.

## Conclusions

The combination of the functionality of organic polymers, in particular those with stimuli‐responsive properties, with a stable mesoscale pore system represents a major challenge. We have solved this problem by using a sol‐gel precursor, which is a monomer at the same time: a styrene‐bridged silsesquioxane compound. Mesoporous organosilica nanoparticles were prepared first. A fraction of the styrene groups imbedded in the surface of the pores were then transformed into radical species as proven by electron paramagnetic resonance (EPR) spectroscopy. These radicals initiate the intrapore polymerization of a second monomer type, which has been added. The organic polymer contains segments of the organosilica and, thus, can be described as a new type of block copolymer. As a result, the organic polymer is tightly attached to the surface of the mesoporous matrix and covers it. Leaking as well as pore blocking does not occur.

Another advantage of an organosilica material containing a high density of vinyl groups, such as the case presented here, is the entry into click chemistry, for instance the thiol‐ene reaction. We used this method to prepare core‐shell nanoparticles. The polymer attached to the shell poly(*N*‐isopropylacrylamide) differed from the one incorporated in the pore system (pentafluorostyrene). The unique combination of porosity, hydrophobic confinement, and thermoresponsive shell allowed the removal of hydrophobic compounds from water, for instance, hormones. The compound is taken from solution, pores were closed, eventually aggregation of the particles takes place, particles are separated, and finally the entrapped species is released again. The particles presented here have a capacity of 69.4 mg per g of the particles and a release efficiency of around 96 %. In the future, the particles could be adapted to specific impurities due to their high flexibility regarding their inner and outer functionalization. Attention should be paid to the fine balance regarding the affinity of the polymer inside the pores to the guest species. Is this affinity very high, there is quantitative removal from the wastewater but also only partial release from nanoporous organosilica particles. Is the affinity weak, clean particles are obtained after release, but waste compounds will have remained in water. Further work needs to address this issue, and the option to attach different polymers with different properties is a promising way to solve this problem.

## Experimental Section

**General information**. Unless stated differently, synthesis was performed using general Schlenk techniques under argon atmosphere. The solvents were dried according to the standard literature^92^ and stored under argon. All starting materials used for synthesis were purchased from commercial sources (Merck or ABCR chemicals). 1,3‐bis(tri‐iso‐propoxysilyl)‐5‐bromobenzene was synthesized according to literature.^18^

**Synthesis of 1,3‐bis(tri‐iso‐propoxysilyl)‐styrene (1)**. 572 mg (13.5 mmol, 3 eq.) lithium chloride and 157 mg (0.22 mmol, 0.05 eq.) bis(triphenylphosphane)palladium(II) dichloride were dissolved in 60 mL dry dimethylformamide and stirred for 10 min. 2.54 g (4.5 mmol, 1 eq.) of 1,3‐bis(tri‐iso‐propoxysilyl)‐5‐bromobenzene were added and the reaction mixture was stirred for further 5 min. 1.7 g (4.5 mmol, 1.2 eq.) of tributyl(vinyl)tin was added dropwise over a period of 5 min before the reaction mixture was heated to 95 °C for 16 h. The reaction was quenched by adding 20 mL water and after cooling the reaction mixture was washed thrice with 50 mL diethyl ether each. The organic phases were combined, dried with magnesium sulfate and the solvent was removed under reduced pressure. The raw product was then purified by ball tube distillation at 110 °C and 5^.^10^−2^ mbar. 1.84 g (3.5 mmol) of **1** were obtained as colorless oil in 96 % yield.

^1^H NMR (400 MHz, CDCl_3_): *δ*=7.90 (t, J=1.2 Hz, 1H), 7.77 (d, J=1.2 Hz, 2H), 6.75 (dd, J=17.6, 10.9 Hz, 1H), 5.76 (dd, J=17.6, 1.0 Hz, 1H), ), 5.24 (dd, J=10.8, 1.0 Hz, 1H), 4.26 (sept, J=6.1 Hz, 6H), 1.21 ppm (d, J=6.2 Hz, 36H). ^13^C NMR (101 MHz, CDCl_3_): *δ*=141.21, 137.45, 135.78, 134.59, 132.16, 113.58, 65.53, 25.63 ppm (‐CH‐(CH_3_)_2_).

**Synthesis of nanoporous nanoparticles from 1,3‐bis(tri‐iso‐propoxysilyl)‐5‐styrene (2)**. 100 mg (0.2 mmol, 1 eq.) of the precursor were dissolved in 1.0 mL 2‐propanol in a screw cap glass. To initiate hydrolysis 0.62 mL of a 0.01 M hydrochloric acid solution were added under stirring (700 rpm) and the turbid reaction mixture was stirred for 3 h at RT (700 rpm). The resulting clear solution was used for the condensation reaction without further purification.

In a screw cap glass 40 mg (6^.^10^−3^ mmol) Pluronic P123, 40 mg (0.11 mmol) CTAB and 0.43 mmol of 1,3,5‐triisopropylbenzene were dissolved in 2 mL of a 0.1 M carbonate buffer (pH=9.4) for the condensation reaction. The prehydrolyzed precursor species was added in a single shot under vigorous stirring (800 rpm) and the resulting suspension was stirred for 2 d at 25 °C. The colorless solid was separated by centrifugation at 6000 rpm and then washed with ethanol, water, and acetone. To remove the organic template, the solid was dispersed in 20 mL of a 1 : 4 (v/v) solution of concentrated hydrochloric acid and ethanol and stirred for two days at 25 °C. The solid was separated by centrifugation at 6000 rpm and then washed with ethanol, water, and acetone to obtain a colorless powder in 60 % yield.

**Copolymerization of 2 with 2,3,4,5,6‐pentafluorostyrene (3 b)**. 50 mg of **2** were placed in a Schlenk tube and dried under vacuum overnight. 0.4 eq. of the pore volume (for 2 cm^3^ g^−1^: 40 μL, 0.28 mmol, 1.2 eq. (with respect to the precursor) 2,3,4,5,6‐pentafluorostyrene and 150 μL of a 0.08 M AIBN solution in DCM was added. The particles were stored in the dark at 5 °C for 24 h before DCM was removed by a constant flow of nitrogen for 2 h. The impregnated particles were degassed by three freeze‐pump‐thaw cycles and equilibrated for 24 h under static vacuum. 2 mL of degassed chloroform and 67 μL (0.5 mmol, 2 eq.) of 2,3,4,5,6‐pentafluorostyrene were added to the particles and the polymerization was initiated by heating to 60 °C for 16 h followed by heating to 100 °C for 2 h. The resulting particles were washed with chloroform and ethanol each before they were dried in vacuum.

**Shell‐functionalization by thiol‐ene click chemistry**. 50 mg of core‐functionalized NOPS were placed in a quartz cell. 3 mg (0.01 mmol, 0.05 eq.) 2,2‐dimethoxy‐2‐phenylacetophenone and 50 mg (0.02 mmol, 0.08 eq., *M*_W_=3000 g mol^−1^) of thiol‐terminated PNIPAM were dissolved in 3 mL of ethanol. The reaction mixture was degassed by nitrogen bubbling before it was irradiated by a mercury vapor lamp for 12 h. The resulting particles were washed with dichloromethane and ethanol each before they were dried in vacuum.

**Separation of dye molecules from water using multifunctional core‐shell NOPs**. 10 mg of core‐shell NOPS were dispersed in 30 mL of water before 300 μL of a 3.5 mg mL^−1^ Solvent Yellow 14 solution in ethanol was added. For the dye uptake, the reaction mixture was stirred for 6 h before it was heated to 40 °C for 30 min to close the pores. The particles were centrifuged, and the supernatant solution was investigated by UV/Vis spectroscopy to determine the amount adsorbed. Afterwards, the particles were washed rapidly to remove surface bound dye molecules, and make sure the following experiments address only guest molecules confined to the pores. To prove that the pores are sealed in the closed state, the particles were redispersed in warm ethanol (40 °C) and stirred for 6 h. For quantitative analysis samples were taken after 0.5 h, 1 h, 3 h and 6 h, centrifuged and investigated by UV/Vis. For the investigation of the release the temperature was decreased to 20 °C and the particles were stirred for 15 h. Again, samples were taken after 0.5 h, 3 h, 6 h and 15 h centrifuged and investigated by UV/Vis spectroscopy. As a reference, this experiment was also performed with unfunctionalized NOPs. To show the recyclability of the particles the uptake‐release cycle was repeated 5 times and the resulting release efficiencies were compared to the initial concentration. Between each cycle the particles were regenerated by redispersion in ethanol and treatment with ultrasound for 20 min. Afterwards they were dried and reused in the next cycle.

**Separation of the hormone progesterone from water using multifunctional core‐shell NOPs**. 10 mg of core‐shell NOPS were dispersed in 3 mL of water before 30 μL of a 1 mg mL^−1^ progesterone in ethanol solution was added. The reaction mixture was stirred for 2 h before it was heated to 40 °C for 30 min. The particles were centrifuged, and the supernatant solution was characterized by high performance liquid chromatography (HPLC). For the investigation of the release the remaining particles were redispersed in ethanol at 20 °C and stirred for 6 h before they were centrifuged at 6000 rpm for 15 min. Again, the supernatant solution was investigated by HPLC‐method.

**Analytical methods**. Attenuated total reflection (ATR) ‐IR Spectroscopy was performed on a Perkin Elmer Spectrum 100 spectrometer with an ATR unit. All spectra were background corrected and normalized to the Si−O−Si vibration at 1044 cm^−1^. Raman spectroscopy was performed on a Perkin Elmer Raman Station 400. N_2_ physisorption measurements were performed with the Micromeritics Tristar 3020 at −196 °C. Prior to analysis the samples were degassed at 85 °C for 720 min. Thermogravimetric Analysis measurements were performed under O_2_ using a Netzsch STA F3 Jupiter with a heating rate of 5 K min^−1^ until 300 °C and 10 K min^−1^ for 300–1000 °C. NMR measurements (^1^H, ^13^C) were performed on a Varian INOVA 400 MHz spectrometer. Magic‐angle spinning (MAS)‐NMR spectra were recorded using a Bruker DRX 400 spectrometer. Spectra were recorded with a spinning frequency of 10000 Hz and a relaxation delay of 120 s. Scanning electron microscopy was performed on a Zeiss Auriga Crossbeam microscope. Liquid chromatography was measured with Thermo Fisher Scientific Dionex 3000. As the column, Agilent Poroshell 120 EC−C18 (2.1×100 mm, 2.7 μm) was used. UV/Vis spectroscopy was performed on a Varian Cary 100. The DLS measurements were done by using a Malvern Zen5600. SI‐MS data were acquired on a Bruker microtof II system. The solutions were injected directly into the evaporation chamber. TEM was acquired on a Zeiss Libra 120 system. HRTEM images have been recorded on a JEOL JEM‐2200FS instrument with an acceleration voltage of 200 kV.

## Conflict of interest

The authors declare no conflict of interest.

## Supporting information

As a service to our authors and readers, this journal provides supporting information supplied by the authors. Such materials are peer reviewed and may be re‐organized for online delivery, but are not copy‐edited or typeset. Technical support issues arising from supporting information (other than missing files) should be addressed to the authors.

Supplementary
