# Supplementary material for: Copolymerization of Mesoporous Styrene‐Bridged Organosilica Nanoparticles with Functional Monomers for the Stimuli‐Responsive Remediation of Water
Source: ChemSusChem. 2020 Aug 10;13(18):5100–11. doi: 10.1002/cssc.202001264 (PMC7540170; doi:10.1002/cssc.202001264)
Supplement: Supplementary file 1 — Supplementary [file CSSC-13-5100-s001.pdf]

ChemSusChem

Supporting Information

Copolymerization of Mesoporous Styrene-Bridged Organosilica Nanoparticles with Functional Monomers for the Stimuli-Responsive Remediation of Water**

Dennis Kollofrath, Marcel Geppert, and Sebastian Polarz*© 2020 The Authors. Published by Wiley-VCH GmbH. This is an open access article under the terms of the Creative Commons Attribution License, which permits use, distribution and reproduction in any medium, provided the original work is properly cited.

Fig. S1. Characterization of the sol-gel precursor (**1**).

(a) ^{1}H-NMR

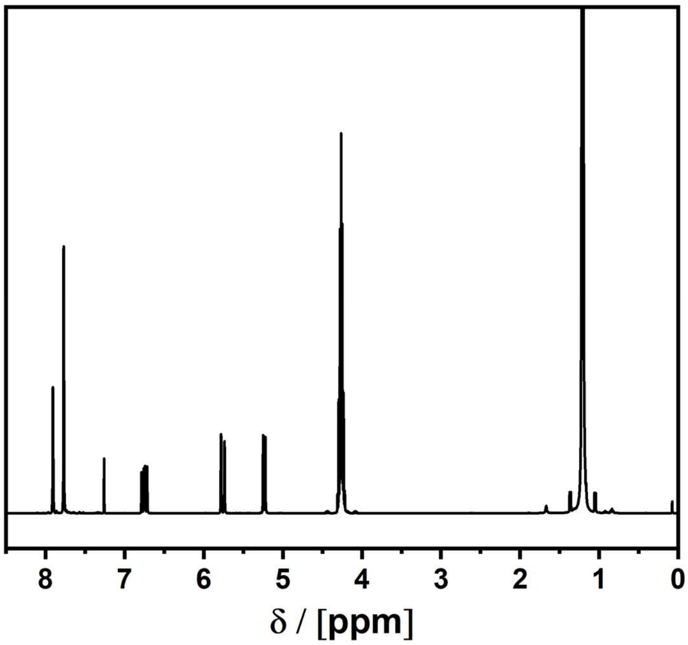

^{1}**H**-NMR (400 MHz, $CDCl_3$): δ = 7.90 (t, *J* = 1.2 Hz, 1H, *p*-arom. **H**), 7.77 (d, *J* = 1.2 Hz, 2H, *o*-arom **H**), 6.75 (dd, *J* = 17.6, 10.9 Hz, 1H, -**CH**=CH_2), 5.76 (dd, *J* = 17.6, 1.0 Hz, 1H, -CH=CH**H'**),), 5.24 (dd, *J* = 10.8, 1.0 Hz, 1H, -CH=C**H**H'), 4.26 (sept, *J* = 6.1 Hz, 6H, -O-**CH**-$(CH_3)_2$), 1.21 (d, *J* = 6.2 Hz, 36H, -O-CH-($\mathbf{CH_3}$)$_2$).

(b) ^{13}C-NMR

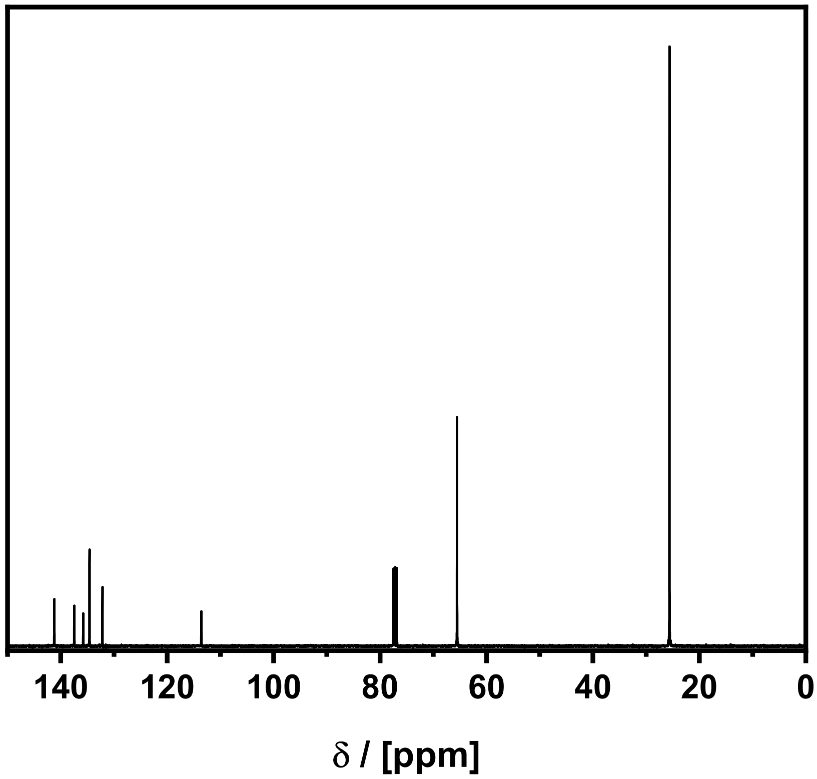

^{13}C NMR (101 MHz, $CDCl_3$): δ(ppm) **=** 141.21 (*p*-arom. **C**), 137.45 (-**CH**=CH_2), 135.78 (Si-arom. **C**), 134.59 (*o*-arom. **C**), 132.16 (-**C**-CH=CH_2), 113.58 (-CH=**CH_2**), 65.53 (-**CH**-$(CH_3)_2$), 25.63 (-CH-(**CH_3**)$_2$).

(c) ESI-MS

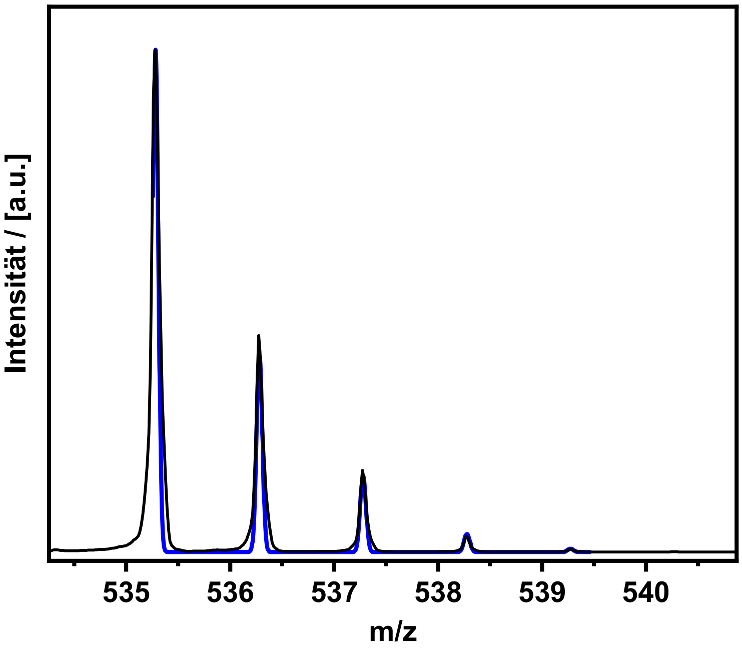

m/z: 535.30 (100.0%), 536.30 (38.3%), 537.30 (14.6%), 538.30 (1.9%)

Fig. S2. Hydrolysis conditions of the sol-gel precursor (**1**).

(a) Hydrolysis of -Si(O^{i}Pr)$_3$ groups.

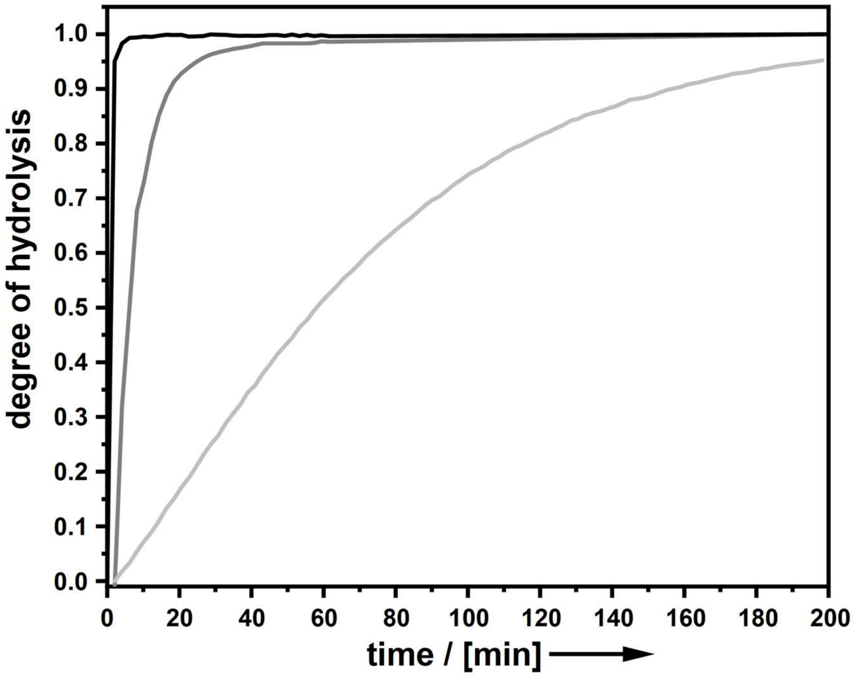

In an NMR tube, 45 mg (0.09 mmol) of the precursor is dissolved in 0.5 mL 2-propanol-d8. Then 0.3 mL of a 1 M hydrochloric acid solution are added and the solution is mixed by turning the tube over. At intervals of 2 min, ^{1}H-NMR spectra are recorded until the hydrolysis is finished. The spectra were evaluated using the septett of the isopropyl groups attached to the silicon atom the free isopropanol.pH = 2 (light grey); pH = 1 (dark grey); pH = 0 (black).

(b) Stability of the vinyl group.

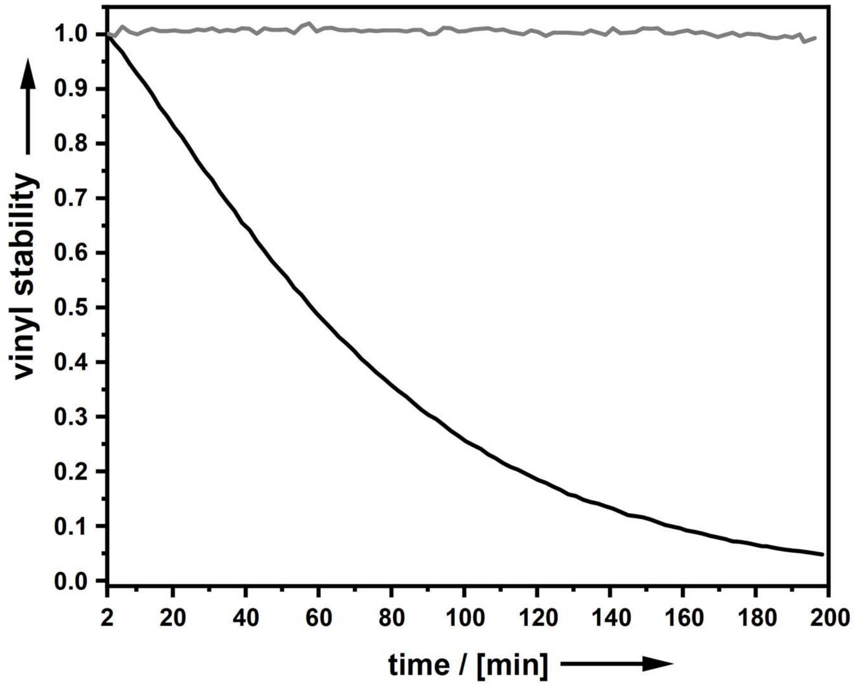

The stability of the vinyl group was investigated by NMR-spectroscopy following the changes in the signals, which are characteristic for C=C (see Fig. S1). pH =1 (grey); pH = 0 (black).

Fig. S3. Additional analytical information to material (**2**)

a) Raman spectra of material (**2**) and the molecular precursor as a reference

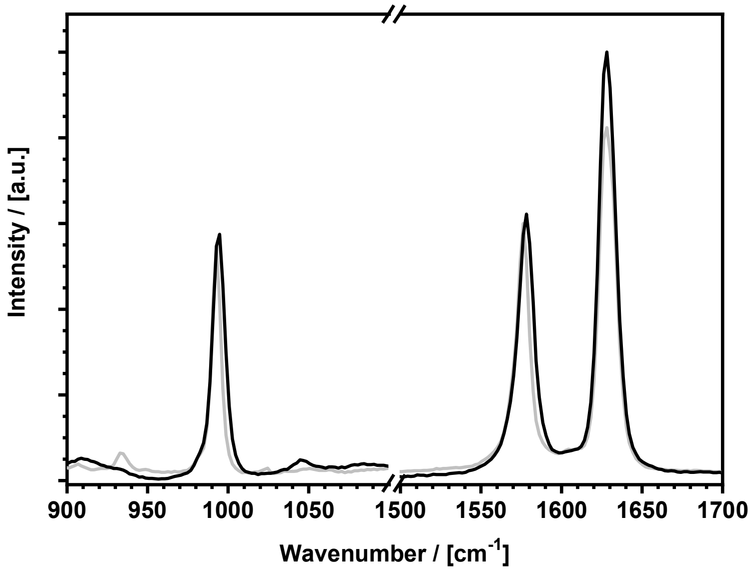

black = precursor (2)
Grey = material

b) ^{13}C-MAS-NMR spectrum of (**2**) (HPDEC)

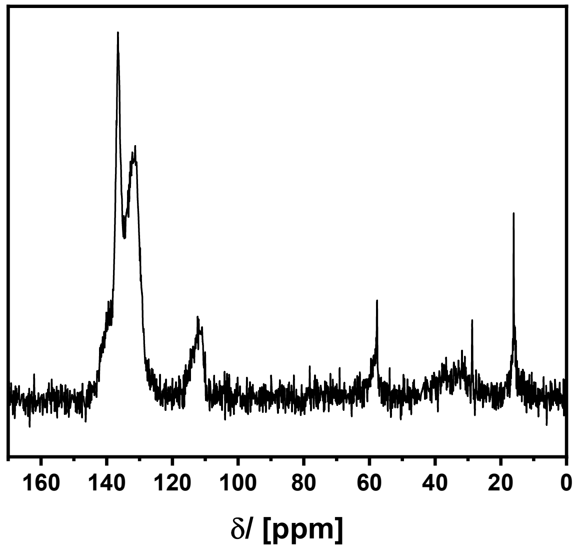

The signals of the phenyl ring (122-140 ppm) are distinct from the vinyl signals (115 ppm). Evaluating the signal intensities demonstrates that ≈80% of the vinyl groups are still present. Signals at 18 ppm and 60 ppm can be assigned to remaining ethanol in the pores

c) ^{1}H-NMR-spectrum of material (**2**) dissolved with NaOD

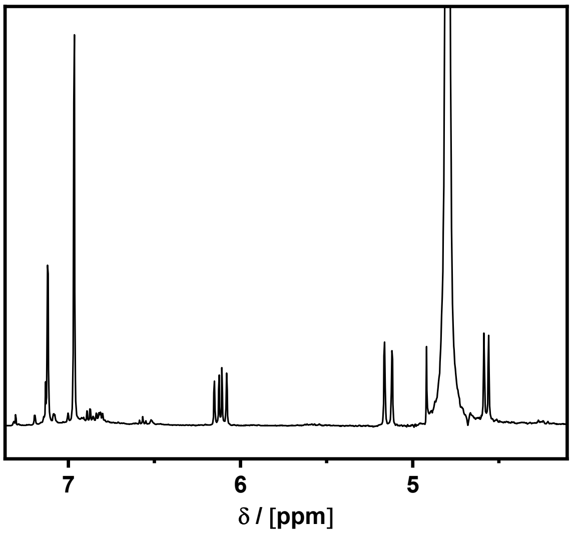

Material (**2**) is dissolved in an aqueous solution of 1M NaOD and a ^{1}H-NMR spectrum was measured. The signals at δ = 7.90 and 7.77 correspond to the aromatic Hs while the protons at δ 6.75, 5.76 and 5.24 can be assigned to the vinyl group of the molecule. The comparison of the signal intensity of the aromatic protons to those of the vinyl function shows that nearly every vinyl group is still intact after extraction.

d) TGA measurement of material (**2**) taken in air.

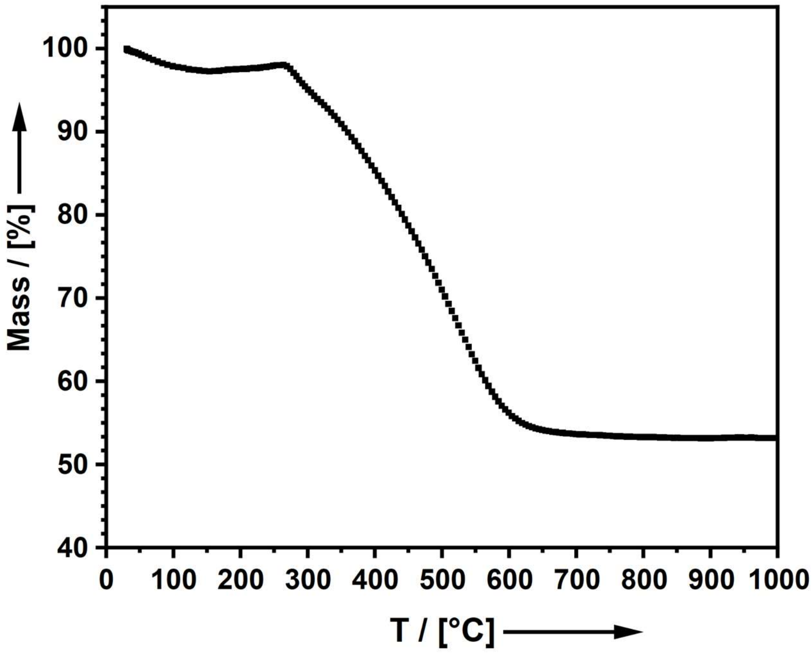

e) comparison of Pluronic P123 and Pluronic 31R1 as a template

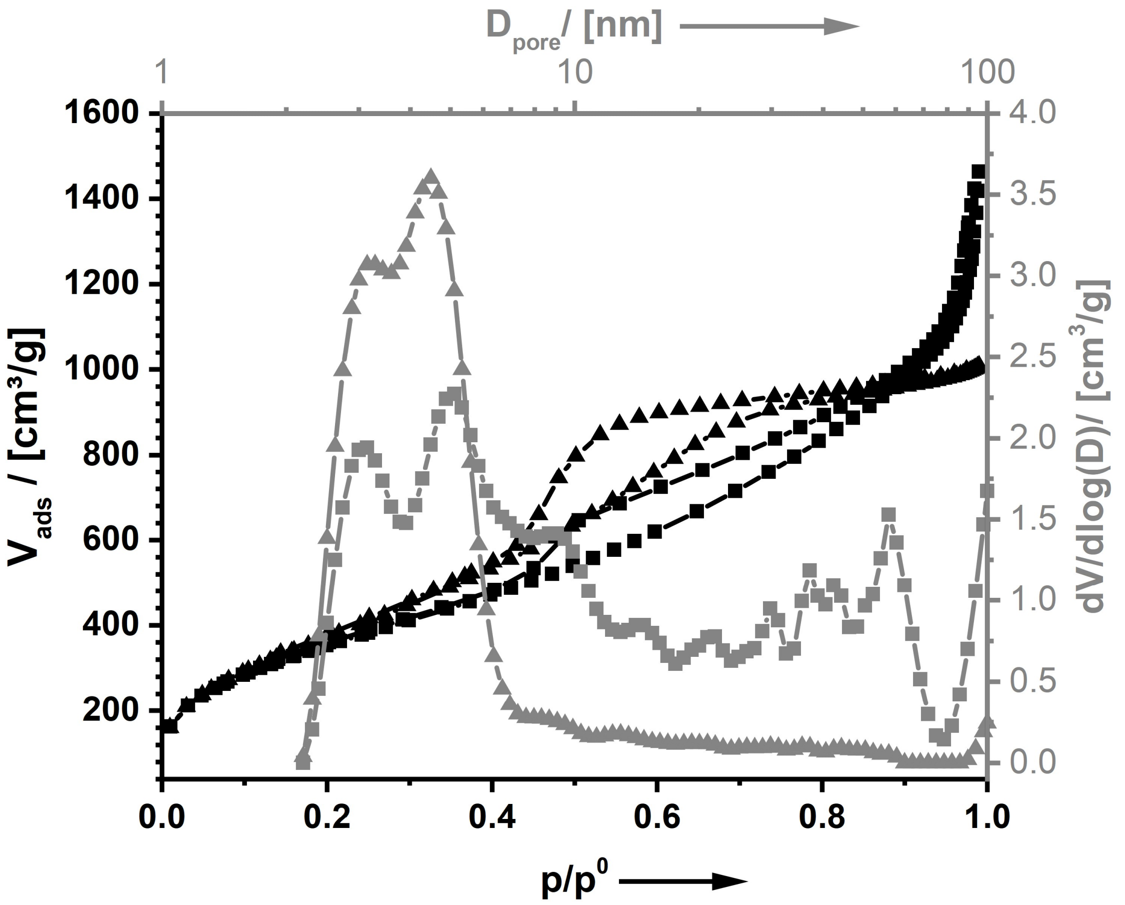

black: isotherms (triangle: 31R1, square: Pluronic P123)
grey: pore size distribution (triangle: 31R1, square: Pluronic P123)

Fig. S4. Polymerization of polystyrene outside of the NOPs.

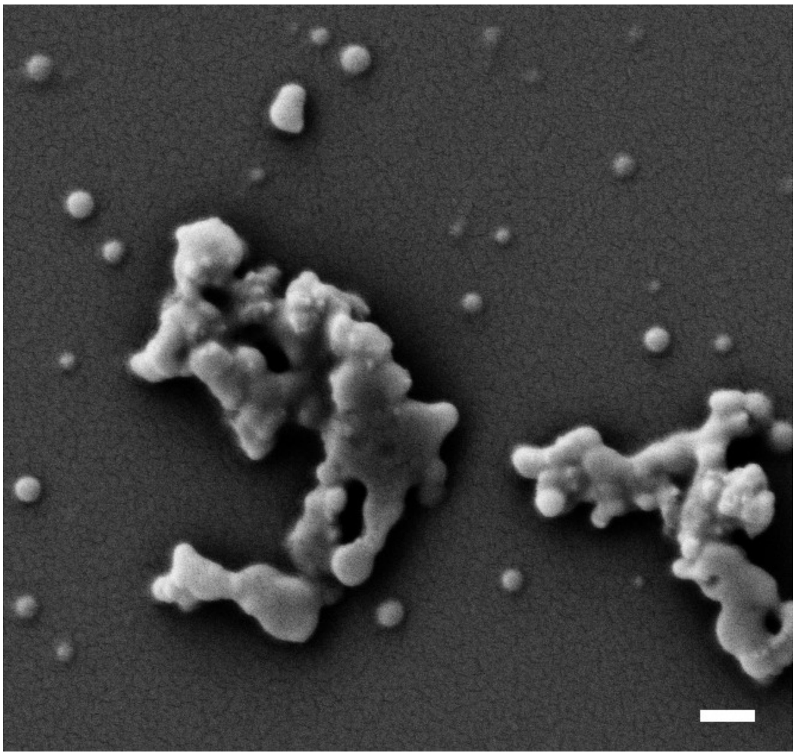

SEM micrograph (scalebar = 200nm).

Fig. S5. Comparison of NOPs before (materials **2**) and after copolymerisation with perfluorostyrene (material **3b**).

(a) SEM (scalebars = 200nm)

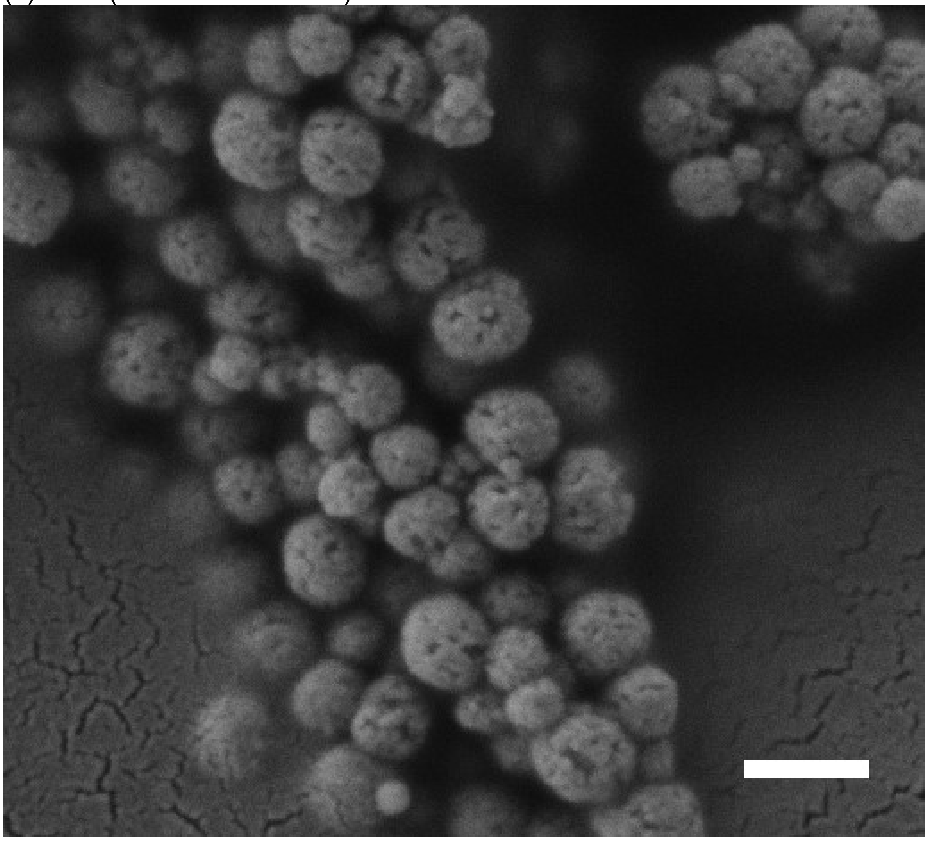

BEFORE

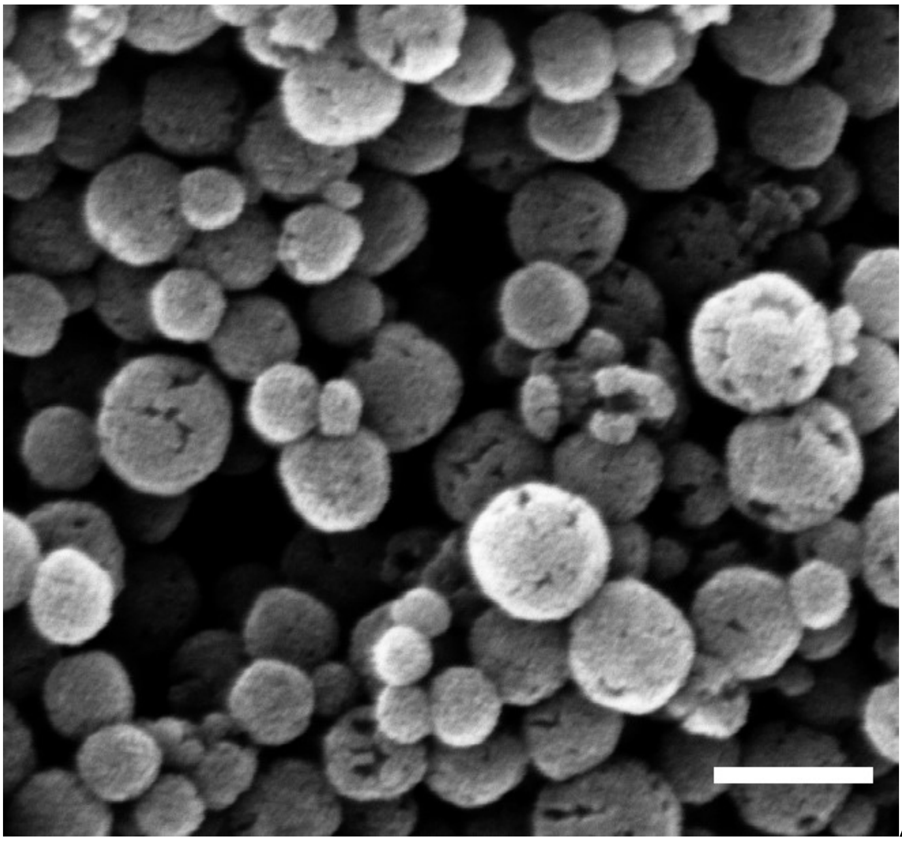

AFTER

(b) TEM (scalebars = 200nm)

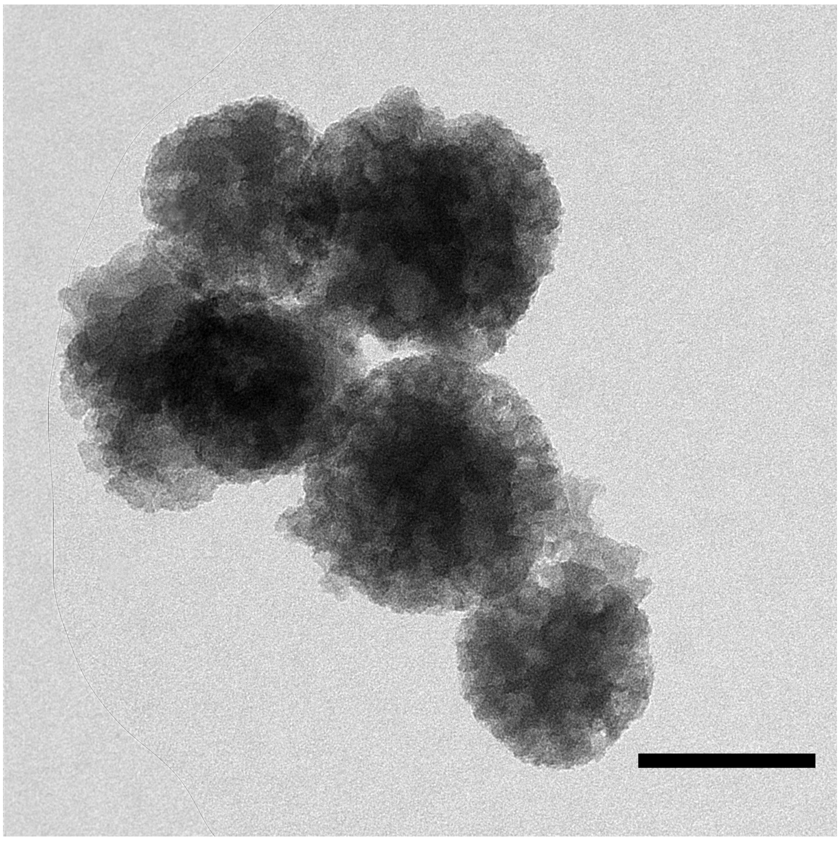

BEFORE

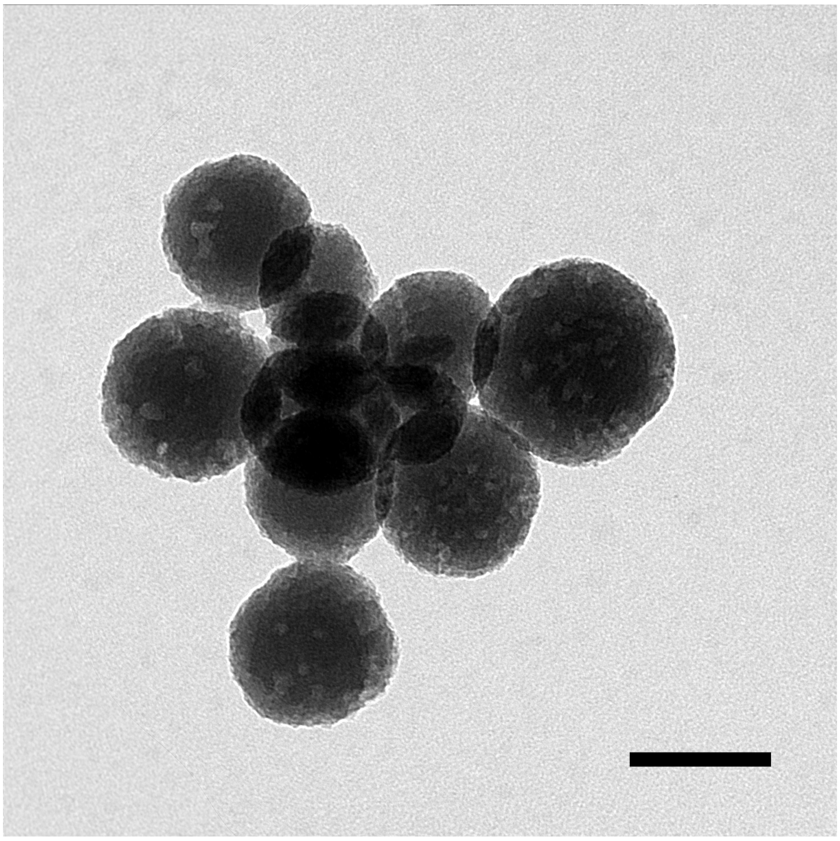

AFTER

(c) IR-spectroscopy

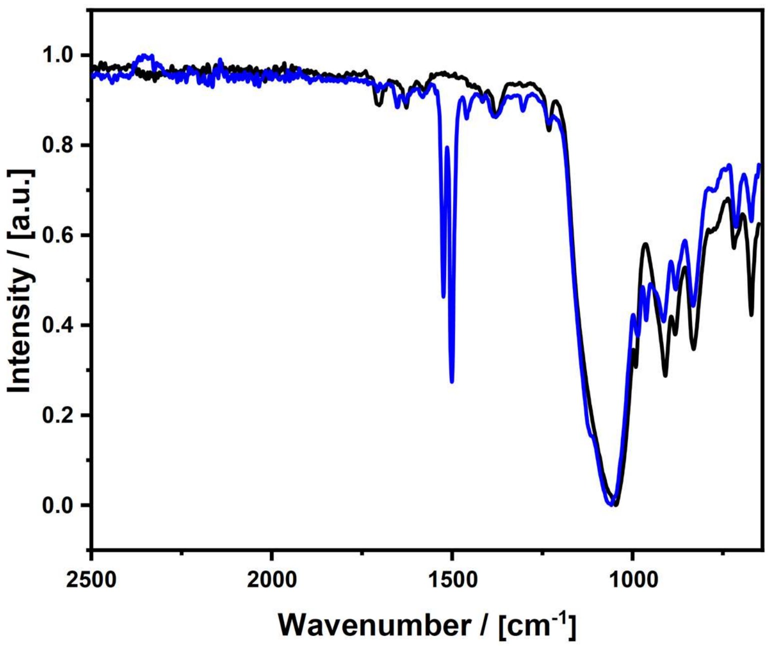

black = material **2**
blue = material **3b**

(d) Pore-size distribution function derived from N_2-physisorption measurements

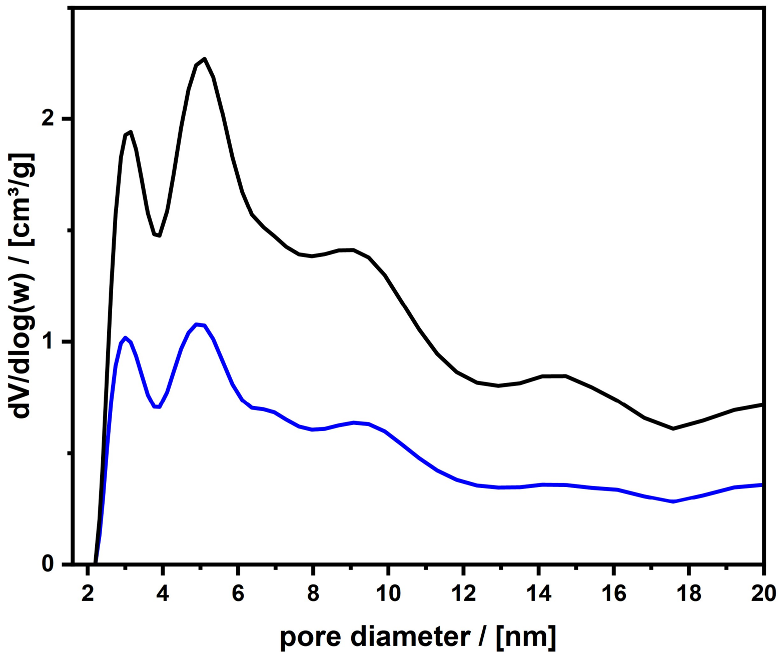

black = material **2**
blue = material **3b**

Fig. S6. Functionalization of the pore system by copolymerization with different monomers

a) EDX-spectra of NOPs functionalized with different monomers

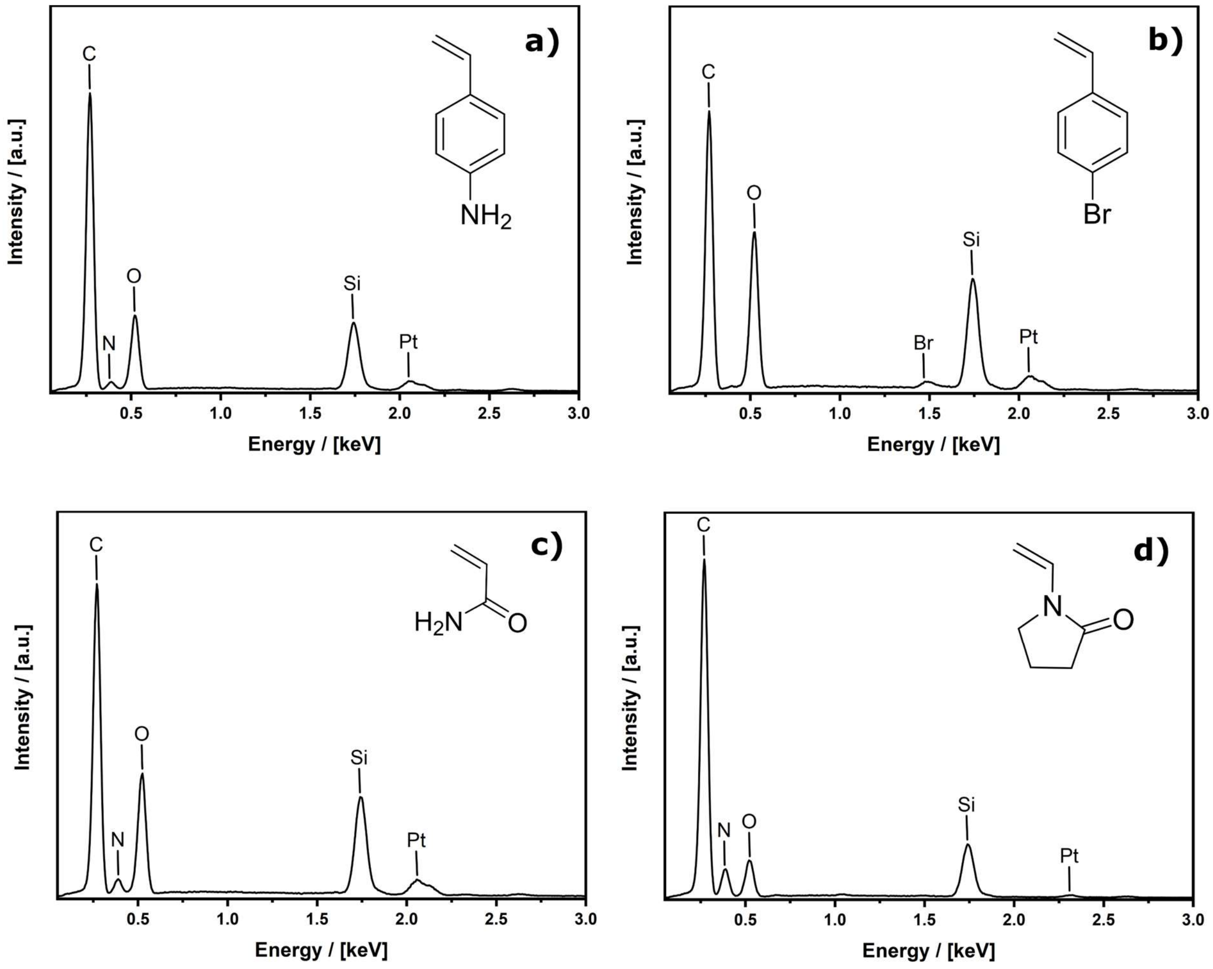

b) Isotherms and pore size distributions derived from N_2-physisorption measurements

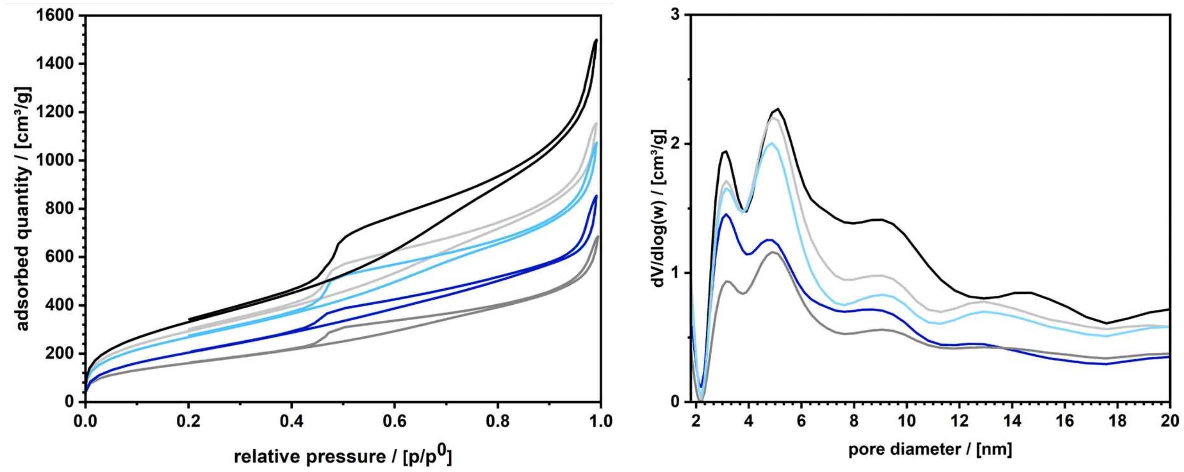

black = unfunctionalized NOPS

light grey = 1-bromo-4-vinylbenzene modified NOPs

light blue = 4-vinylaniline modified NOPs

dark blue = 1-vinylpyrrolidin-2-one modified NOPs

dark grey = acrylamide modified NOPs

Fig. S7. Additional analytical data for the pentafluorostyrene core, PNIPAM-shell NOPS

a) Isotherms derived from N_2-physisorption measurements

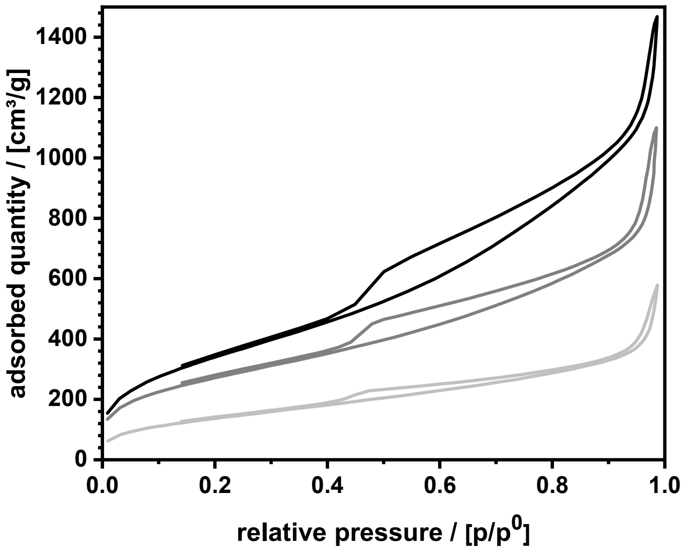

black = unfunctionalized NOPS
dark grey = NOPS after core-functionalization with perfluorostyrene
grey = NOPS after shell functionalization with PNIPAM

b) Pore-size distribution function derived from N_2-physisorption measurements

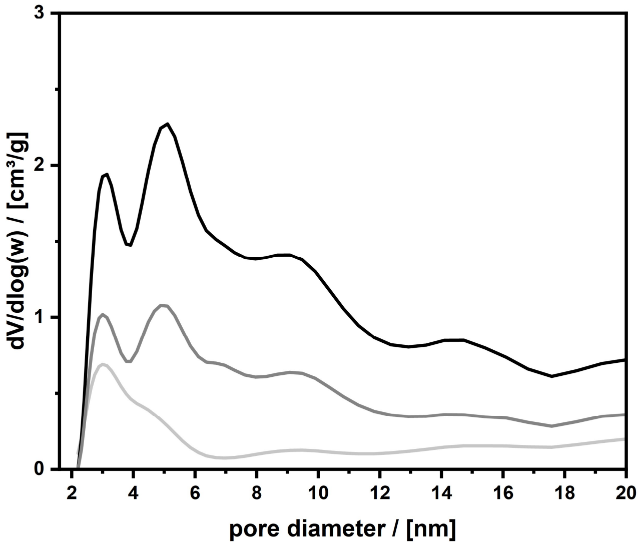

Black = unfunctionalized NOPS
dark grey = NOPS after core-functionalization with perfluorostyrene
grey = NOPS after shell functionalization with PNIPAM

Fig. S8 UV/Vis spectra to the Solvent Yellow 14 release experiments

a) Regression line for the quantitative analysis of the release process

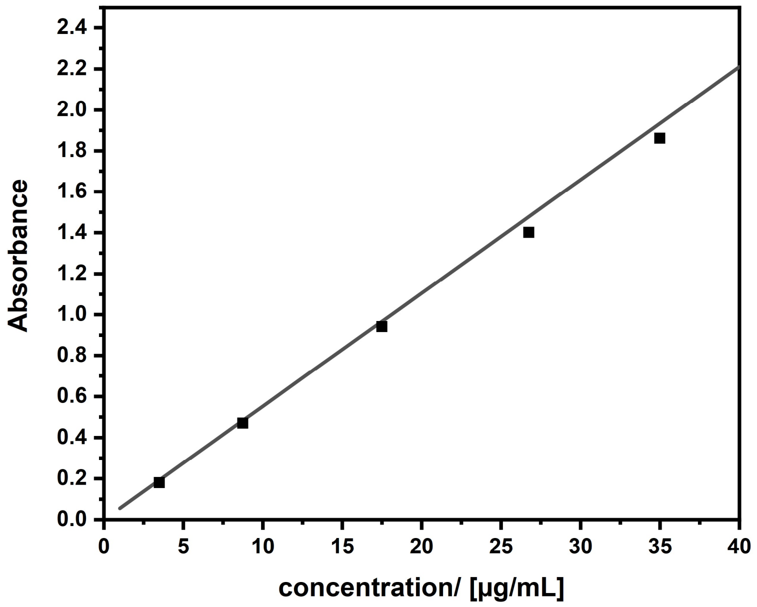

b) UV/Vis spectra of SY 14 release using core-shell NOPs with PNIPAM shell

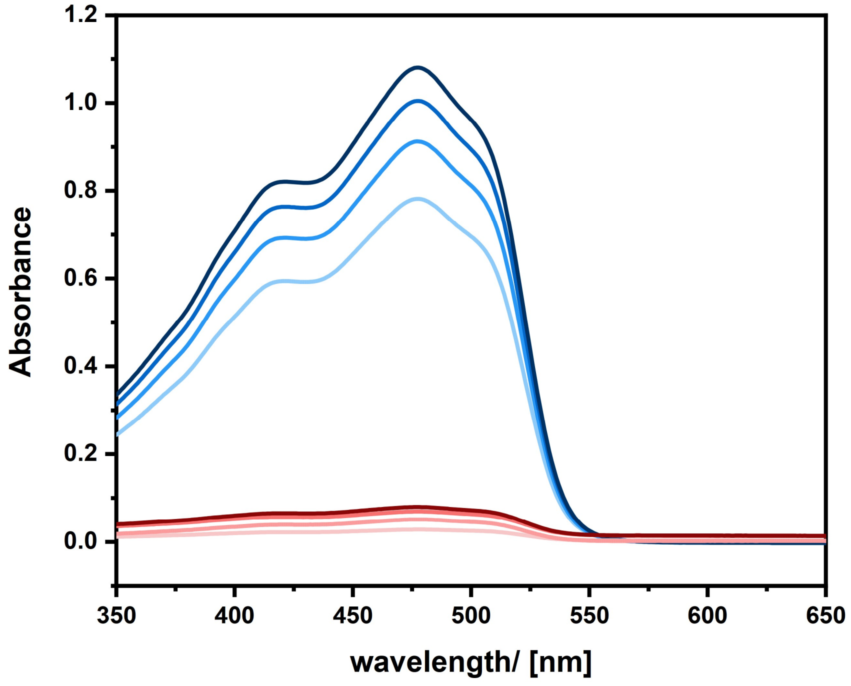

Red = Release at 40 °C (From top to buttom: after 6h, 3h, 1h, 0.5h)
Blue = Release at 20°C (From top to buttom: after 15h, 6h, 3h, 0.5h)

Calculation of the capacity

$$q_m = \frac{(c_E + c_L)V}{W} \quad (1)$$

q_m= capacity [mg/g]
c_L= concentration of the possibly leaked dye solution (Lambert Beer)
c_E= end concentration of the dye solution after release (Lambert Beer)
V = Volume of the dye solution (30 mL)
W = Mass of the particles used (0.01 g)

Calculation of the release efficiency

$$\%\,released = \frac{(c_{eff} - c_E)}{c_{eff}} * 100 \quad (2)$$

c_{eff}= (c_0-c_w)
c_0 = initial concentration of dye solution (3.5 mg/mL)
c_W = concentration of the washing solution
c_E= end concentration of the dye solution after release (Lambert Beer)
V = Volume of the dye solution (30 mL)
W = Mass of the particles used (0.01 g)

c) Comparison of the dye uptake of unfunctionalized NOPs and PNIPAM functionalized NOPS.

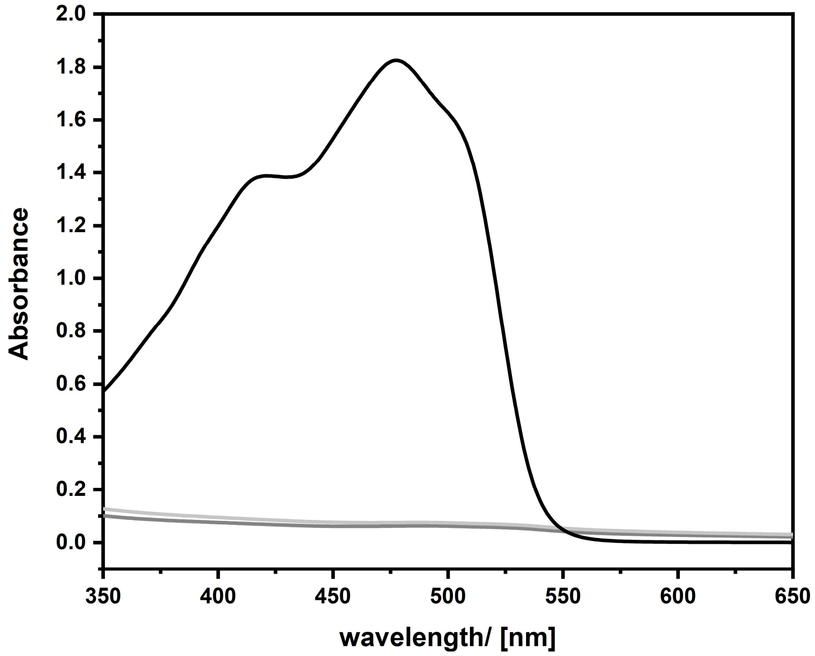

d) UV/Vis spectra of SY 14 release using core-shell NOPs without PNIPAM shell.

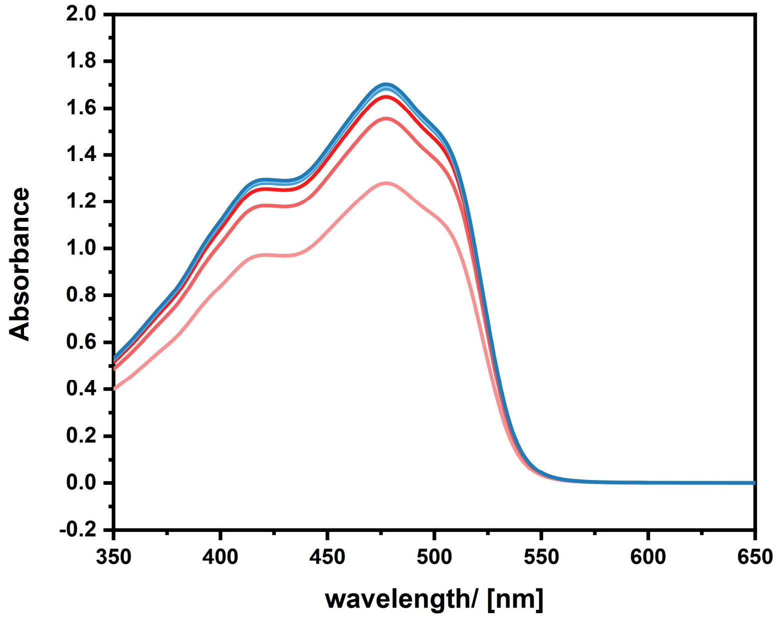

Red = Release at 40 °C (From top to buttom: after 6h, 3h, 1h, 0.5h)
Blue = Release at 20°C (From top to buttom: after 15h, 6h, 3h, 0.5h)
